# Molecular imaging can identify the location to perform a frozen biopsy during intraoperative frozen section consultation

**DOI:** 10.1371/journal.pone.0252731

**Published:** 2021-06-04

**Authors:** Mitchell G. Bryski, Lydia G. Frenzel-Sulyok, E. James Delikatny, Charuhas Deshpande, Leslie A. Litzky, Sunil Singhal

**Affiliations:** 1 Department of Surgery, Perelman School of Medicine, University of Pennsylvania, Philadelphia, Pennsylvania, United States of America; 2 Department of Radiology, Perelman School of Medicine, University of Pennsylvania, Philadelphia, Pennsylvania, United States of America; 3 Department of Pathology & Laboratory Medicine, Perelman School of Medicine, University of Pennsylvania, Philadelphia, Pennsylvania, United States of America; 4 Abramson Cancer Center, Perelman School of Medicine, University of Pennsylvania, Philadelphia, Pennsylvania, United States of America; Stanford University, UNITED STATES

## Abstract

**Background:**

Intraoperative frozen section (FS) consultation is an important tool in surgical oncology that suffers from sampling error because the pathologist does not always know where to perform a biopsy of the surgical specimen. Intraoperative molecular imaging is a technology used in the OR to visualize lesions during surgery. We hypothesized that molecular imaging can address this pathology challenge in FS by visualizing the cancer cells in the specimen in the pathology suite. Here, we report the development and validation of a molecular-imaging capable cryostat called Smart-Cut.

**Methods:**

A molecular imaging capable cryostat prototype was developed and tested using a murine model. Tumors grown in mice were targeted with a NIR contrast agent, indocyanine green (ICG), via tail vein injection. Tumors and adjacent normal tissue samples were frozen sectioned with Smart-Cut. Fluorescent sections and non-fluorescent sections were prepared for H&E and fluorescent microscopy. Fluorescent signal was quantified by tumor-to-background ratio (TBR). NIR fluorescence was tested in one patient enrolled in a clinical trial.

**Results:**

The Smart-Cut prototype has a small footprint and fits well in the pathology suite. Fluorescence imaging with Smart-Cut identified cancerous tissue in the specimen in all 12 mice. No false positives or false negatives were seen, as confirmed by H&E. The mean TBR in Smart-Cut positive tissue sections was 6.8 (SD±3.8). In a clinical application in the pathology suite, NIR imaging identified two lesions in a pulmonary resection specimen, where traditional grossing only identified one.

**Conclusion:**

Molecular imaging can be integrated into the pathology suite via the Smart-Cut device, and can detect cancer in frozen tissue sections using molecular imaging in a murine model.

## Introduction

Intraoperative frozen section (FS) consultation is a valuable tool in surgical oncology to rapidly obtain data that will affect the patient’s operative plan. Most commonly, FS is used to evaluate margin status, identify benign versus malignant tissue and lymph nodes, or classify tumors [1]. FS consultation is particularly important in thoracic oncology, where almost half of patients undergo pulmonary resection for a suspicious lung mass without a pre-operative tissue biopsy [2].

One of the challenges during FS is identifying where the cancer cells are located in a surgical specimen. The gross appearance of the specimen may be clouded by blood and surgical manipulation. The specimen may contain granulomas, inflammation, and cancer cells. The goal of the FS is to tease out the cancer cells in the specimen and avoid a false positive reading by missing the actual neoplasm. Tissue sampling error does occur when the pathologic tissue is present only in deeper sections of the FS block not reviewed during intraoperative FS consultation. These sampling errors result from an inability to identify or visualize pathologic tissue during grossing and sectioning [3]. Despite the important information that needs to be generated, the operation is effectively put on hold while awaiting the results of FS consultation, thus speed is also another important consideration for patient safety, with most FS results reported back to the OR within 20–30 minutes [4].

Over the last 5 years, a new approach called Intraoperative Molecular Imaging (IMI) has been used effectively by surgeons to visualize pathologic lesions during surgery. For IMI, a fluorescent contrast agent is injected into the patient prior to surgery and localizes to the target lesion. During surgery, a light source is used to excite the optical contrast agent in the organ of interest and the resulting optical emission is detected with a camera system. IMI allows surgeons to clearly visualize lesions that are invisible to the naked eye, imperceptible on palpation, or even radiographically occult. IMI requires a suitable fluorescent contrast agent, ideally with emission in the NIR range (700–900 nm), and a specialized imaging system to quantify the dye [5]. The fluorescent dye can localize to the tumor based on its comparatively leaky vasculature via the Enhanced Permeability and Retention (EPR) Effect [6], or by actively targeting tumor markers through receptor-ligand binding [7], or antibody affinity [8]. By conjugating a fluorophore to a ligand or antibody targeting tumor-specific cell markers, dyes can localize to the tumor for sensitive and specific visualization, adding a new tool to the surgeon’s arsenal beyond mere visual inspection and manual palpation [4, 5, 9]. Once the dye has localized to the tumor (a process that can take minutes or days, depending on the pharmacokinetics of the dye), it can be excited with the specialized imaging system to make the tumor glow.

We postulated that for patients undergoing IMI, it may have potential value in the pathology suite to identify the cancer in the specimen. We report here the development and validation of a molecular-imaging capable cryostat, called “Smart-Cut,” that leverages molecular imaging to identify cancer cells on frozen section tissue. We demonstrate that Smart-Cut is compatible with standard pathology workflows, identifies cancer on frozen sections in a murine model, and has potential for translation to clinical applications.

## Materials and methods

### Fluorescence detection system

In this early prototype, a small, portable fluorescence imaging system developed in our lab, named the Small Portable Interchangeable Imager of Fluorescence (SPIIF), was re-purposed for integration into Smart-Cut. The SPIIF has been described in detail previously [10]. Briefly, the SPIIF system is composed of a NIR charge-coupled device (CCD) camera with an articulating light filter and LED light source mounted with a screw and washer system to a weighted platform. We selected the SPIIF due to its small footprint (15 cm x 10 cm) and low weight (less than one kilogram). For our preliminary testing of Smart-Cut, we optimized the SPIIF for imaging with ICG using an LED of 740 nm for excitation and a long pass filter at 800nm for signal collection. However, as an interchangeable imager, the SPIIF can be configured to image other dyes at different wavelengths, for example, fluorescein in the visible range.

For use in the clinical case presentation, a commercially available NIR imaging system (VS3 Iridium, Visionsense, USA) was brought into the surgical pathology suite and used as a proof-of-principle for the Smart-Cut technology.

### Mechanical cryostat system

**Fig 1:**
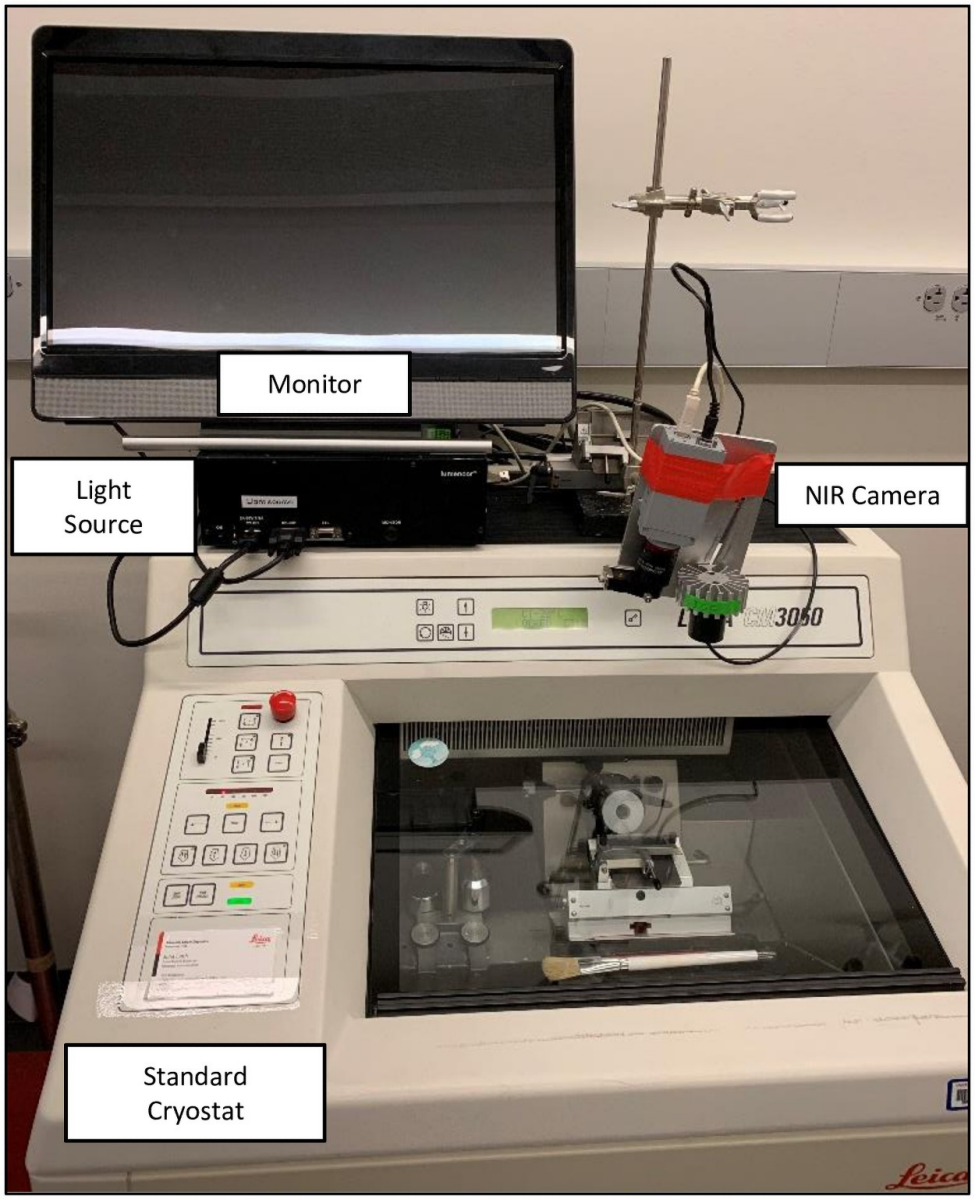
The Smart-Cut prototype. The camera, light, source, and computer from the SPIIF imaging system are mounted to a standard Leica cryostat. The camera head is directed towards the frozen specimen for imaging during sectioning.

The SPIIF imaging system was mounted to a standard research cryostat in our lab (CM3050, Leica Biosystems, Germany). The camera head was directed at the cryochamber, pointing directly at the mounting head and cryostat blade—where the specimen was mounted for sectioning after freezing in OCT (Fig 1).

### Animal experimental model

Twelve female athymic nude mice (Charles River Laboratories, USA) were purchased and housed in pathogen-free conditions and used for experiments at eight weeks of age or older for our flank tumor model. Mice were given normal access to food and water, and maintained on a 12 hour light/dark cycle to minimize animal suffering and distress. The mice were maintained in conditions approved by the Animal Care and Use Committees of the University of Pennsylvania and in agreement with the Guide for the Care and Use of Laboratory Animals. The Institutional Animal Care and Use Committee of the University of Pennsylvania approved all protocols (Protocol #803344).

The KB cell line is a human carcinoma cell line derived from the HeLa cell line, in turn established from human cervical papillomavirus-related (HPV 18) carcinoma [11]. The KB cell line was cultured, maintained, and passaged in RPMI (RPMI 1640 Medium, Gibco Life Technologies) supplemented with 10% fetal bovine serum (FBS; Hyclone), 1% penicillin/1% streptomycin (Gibco Life Technologies) and 1% L-glutamine (Corning). The KB cell line was provided as a gift from Dr. Phil Low, PhD at Purdue University, and regularly tested and maintained negative for Mycoplasma. The cell line was maintained at 37 °C and 5% CO_2_ in a humidified incubator.

Mice were injected subcutaneously with 1x10^6^ KB cells suspended in 100 μL of culture media and Matrigel Matrix in a 1:1 ratio. The tumors were allowed to grow to a volume of 500 mm^3^, approximated by the formula (3.14 x long-axis x short-axis^2^)/6. Mice were then injected with 100 μL of 10 μM ICG through the tail vein as previously described [12]. 24 hours after ICG injection, the mice were euthanized by inhalation of CO_2_ confirmed by cervical dislocation, according to protocol. Mice were euthanized once tumor volume reached 500 mm^3^, and no mice died prior to meeting the criteria for euthanasia. Following euthanasia, mice were imaged on a NIR small animal imaging system (Pearl Trilogy, LI-COR, USA) and the tumors harvested along with adjacent normal muscle tissue.

The tissue was frozen in OCT and sectioned on the Smart-Cut cryostat. Frozen tissue was trimmed until ICG fluorescent signal was detected. Then, two consecutive cuts were mounted on slides (Unifrost Microscope Slides, Azer Scientific, USA), one stained with H&E and one with DAPI (ProLong^™^ Diamond Antifade Mountant with DAPI, Thermofisher, USA), and then imaged under brightfield and NIR light (DM6B Microscope, Leica Microsystems, Germany).

Consecutive cuts of non-fluorescent muscle tissue were also taken as a negative control, and as above, stained with either H&E or DAPI, and imaged for brightfield and fluorescence microscopy. Tumor to background ratios (TBRs) were calculated using region of interest (ROI) analysis comparing fluorescent area to an adjacent non-fluorescent area on ImageJ software (ImageJ 1.50i, NIH, USA).

### Clinical validation

As part of an ongoing clinical trial (NCT02651246) of TumorGlow^®^ for intraoperative identification of cancer, a 45-year-old woman with a history of stage III colon cancer status post hemicolectomy with adjuvant chemotherapy presented to the thoracic surgery clinic for diagnostic biopsy of bilateral lung nodules. The patient provided written informed consent for participation in this clinical trial approved by the University of Pennsylvania Institutional Review Board (IRB #822933–1). She underwent a left VATS wedge resection 24 hours after receiving a 3 mg/kg dose of ICG for intraoperative imaging. Two nodules were resected and sent to the pathology suite for intraoperative FS consultation. Fluorescence imaging of the frozen specimen was conducted in the clinical pathology suite as Smart-Cut proof-of-principle to identify cancer.

## Results

### Workflow

**Fig 2:**
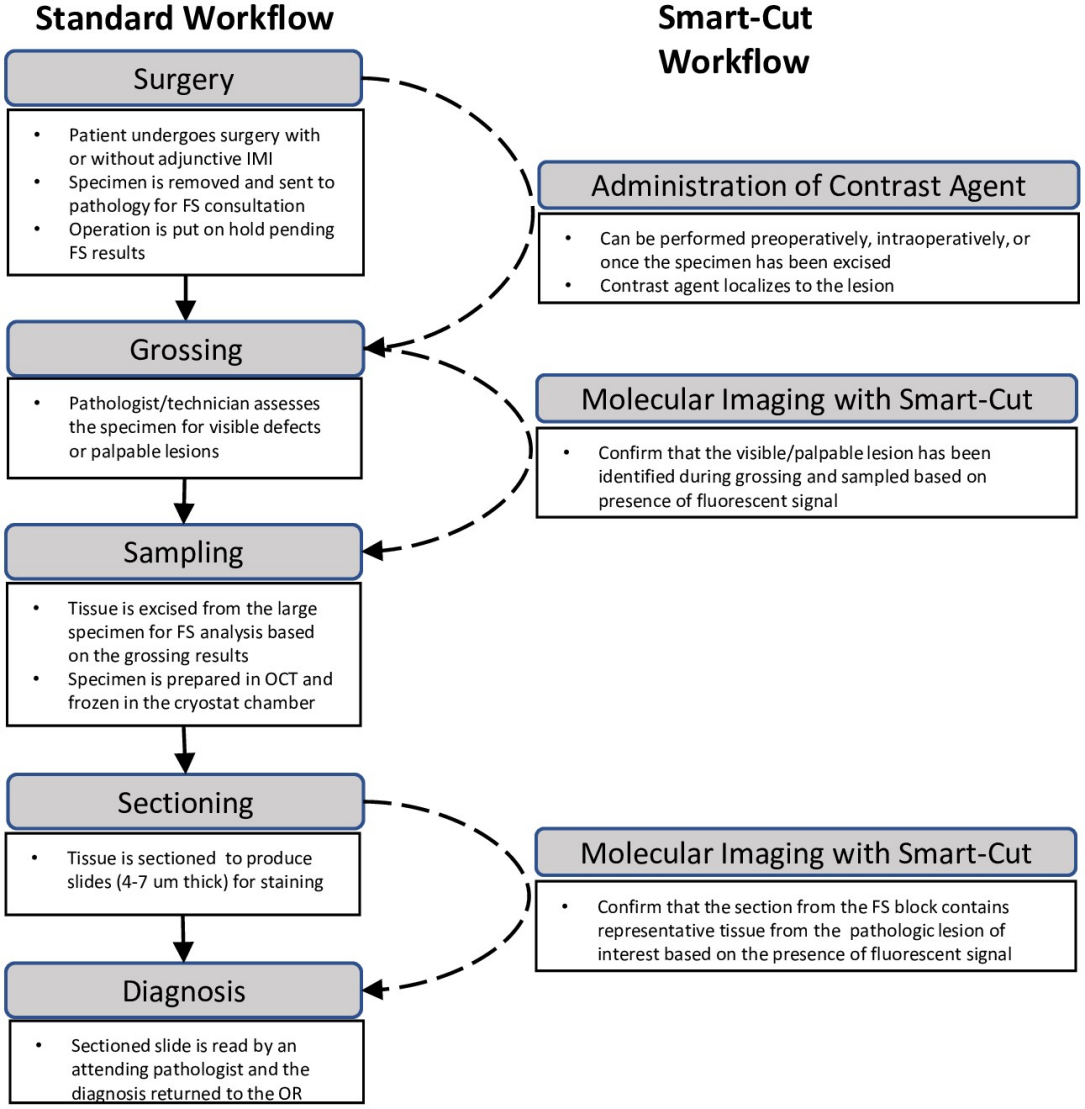
Integration of Smart-Cut into standard pathology workflow.

To ensure Smart-Cut’s potential for broad uptake in frozen section consultation, we designed the device to be easily integrated into the pathology suite with minimal disruption to existing workflows (Fig 2).

**Fig 3:**
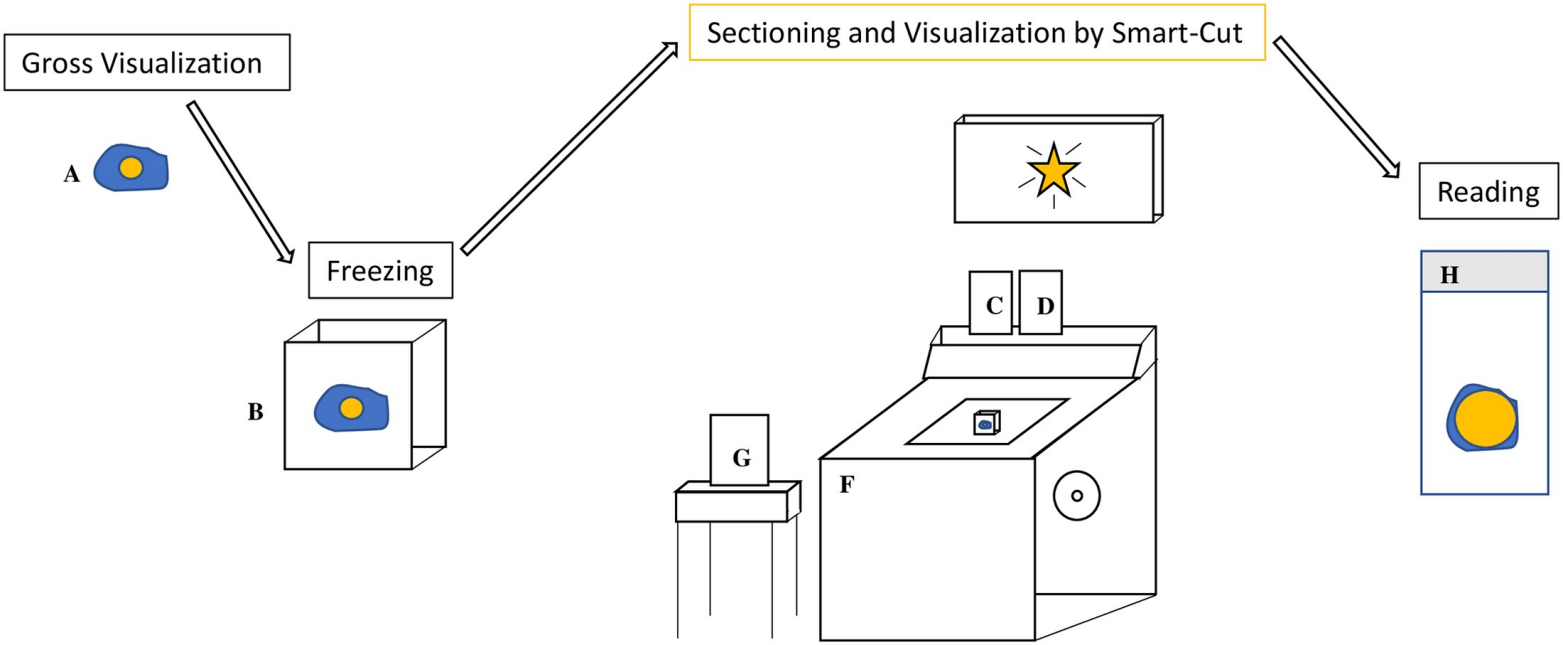
Schematic representation of Smart-Cut workflow. After grossing the specimen (A), either with or without fluorescence imaging by Smart-Cut, the neoplasm is frozen (B) for quick sectioning and fluorescence visualization on the Smart-Cut. Smart-Cut consists of LED light source (C), filter (D), and NIR camera (D) used in conjunction with a standard cryostat (F). Smart-Cut excites a contrast agent localized to the lesion. Emitted light is interpreted by the computer (G) such that changes in detected fluorescence intensity are used to differentiate between cancer and normal tissue. The slide can then be prepared for reading by the pathologist (H). Gold indicates cancer, and blue represents normal tissue; the gold star on the display screen represents fluorescence detected in a tumor section.

The device can be used at multiple steps in the frozen section process to confirm the presence of cancer and is designed as an adjunct to the standard cryostat, an instrument that is currently available in typical pathology suites in hospitals (Fig 3).

The camera system is small and ergonomic (weighing less than 1 kg). The camera’s small footprint (15 cm x 10 cm) allows it to be attached to the cryostat without impinging on its sectioning performance or the comfort of the technician. Additionally, with the interchangeable capabilities of the imaging system, the LED light source and optical filters can be switched out to image a range of fluorescent contrast agents in the visible and NIR ranges without increasing the device footprint. Smart-Cut is easily integrated into existing workflows and instruments of the pathology suite.

### Animal experiments

The Smart-Cut device prototype and proposed workflow were tested in a pre-clinical setting to validate its efficacy. Twelve athymic nude mice with xenograft flank tumors were injected i.v. with ICG [12]. The flank tumors were imaged in the Small Animal Imaging Facility on a Pearl Trilogy (LI-COR, USA) to confirm the presence of the fluorophore within the tumor tissue. All 12 flank tumors were fluorescent with mean TBR 4.4±1.2. The tumor was harvested, frozen in OCT and sectioned on the Smart-Cut. Once fluorescent signal was detected in the OCT block by the Smart-Cut, a 5 μm section was cut, mounted, H&E stained, and imaged on a brightfield microscope (Leica DM6B, Germany). A consecutive slide was then cut and counter-stained with DAPI for fluorescence microscopy. All 12 Smart-Cut fluorescent slides showed cancer present on H&E.

As a negative control, tissue read as non-fluorescent by Smart-Cut was also H&E stained with consecutive cuts counter-stained with DAPI. All 12 slides had normal muscle tissue with no cancer present.

**Fig 4:**
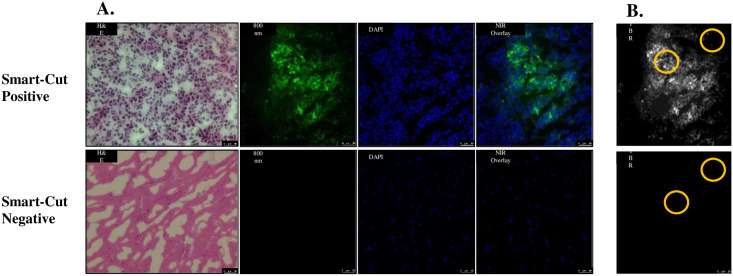
Representative frozen section slides from pre-clinical experiment. (A) “Smart-Cut positive” is fluorescence in tumor, which correlates to the presence of cancer on the H&E. “Smart-Cut negative” is the lack of fluorescence in muscle, which correlates to the absence of cancer on the H&E. DAPI nuclear stain is blue, and the 800 nm NIR fluorescence is pseudo-colored green. All microscopy was done at 20x, and the scale bar is 50 *u*m. (B) NIR 800 nm channel showing positive (top) or negative (bottom) fluorescence. Gold circles indicate areas where mean fluorescence intensity was calculated for TBRs.

Mean TBR of fluorescent to non-fluorescent tissue sections was 6.8±3.8 (Fig 4).

Smart-Cut detected cancer by fluorescence on frozen section with no false negatives or false positives in a pre-clinical murine model.

### Clinical validation

**Fig 5:**
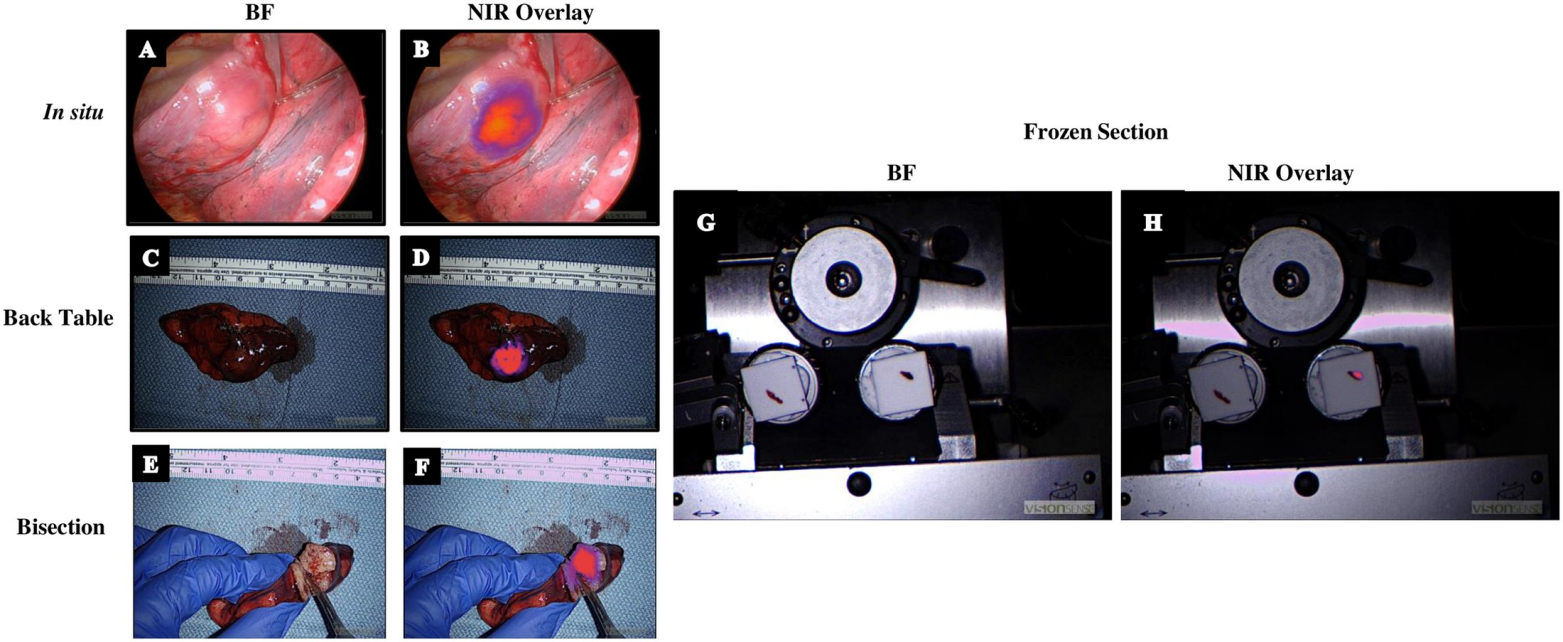
Workflow of surgery with IMI. (A) Traditional VATS visualization of a lesion. (B) IMI performed intraoperatively localizes the target lesion *in situ*. (C) After excision of the specimen, margins can be grossly assessed on the back table and further visualized with IMI (D). Opening the specimen (E) demonstrates lesion-specific uptake of the dye (F). (G) Frozen section samples of uninvolved lung (left) and the cancerous lesion (right). (H) Using IMI, the cancerous lesion can be identified and distinguished from adjacent normal tissue.

The Smart-Cut prototype and workflow were assessed as part of an ongoing clinical trial (NCT02651246) of TumorGlow^®^ for intraoperative identification of cancer. A patient presented for surgical resection of suspected colorectal adenocarcinoma metastases to the lung. Two lesions (one of which was radiographically occult) were identified intraoperatively, as previously reported [13]. The specimens were sent for frozen section pathology, with the suspected lesions mounted in OCT for sectioning. Fluorescence imaging of the specimen on the chuck helped identify the 2 mm occult lesion (TBR = 3.1) (Fig 5). The frozen section cut from this fluorescent tissue was read as colorectal adenocarcinoma and reported back to the OR.

The combination of fluorescence imaging and frozen sectioning embodied in Smart-Cut identifies cancer and has the potential to streamline intraoperative FS consultation.

## Discussion

Intraoperative consultation with frozen section pathology is a valued element of surgical care, but it has several limitations [14]. Chief amongst these limitations is sampling error, which, in a retrospective review of all intraoperative frozen section consultations from a tertiary-level hospital and cancer center during a 6 month period (n = 1042), was responsible for 50% of all discordance between intraoperative consultation with frozen section and final diagnosis [15]. Most typically this occurs because the biopsy was not taken deep enough, or, the concurrent presence of granulomas, inflammation and cancer all existed in the same specimen. Thus, we propose a solution to reduce the occurrence of sampling error by introducing molecular imaging to the pathology suite through Smart-Cut, a cryostat integrated with optical imaging capability that was tested in the pre-clinical and clinical settings.

Smart-Cut has the potential to be broadly used in surgical pathology because of its integrability into existing workflows. With the imaging system’s small footprint incorporated with a standard cryostat, Smart-Cut brings the added benefit of optical imaging to the pathology suite without the additional bulk of traditional back-table imaging devices.

In a pre-clinical murine model, Smart-Cut demonstrated the potential to identify cancer in frozen section tissue with neither false positives nor false negatives. This reassurance from optical imaging can give the pathologist additional confidence that the pathologic lesion has been appropriately sampled with the potential to minimize the incidence of sampling error. Confirmation with fluorescent signal also has the potential to speed up the FS consultation by limiting the need for re-sectioning tissue to confirm the presence of cancer.

Finally, the Smart-Cut was employed in the surgical pathology suite of a high-volume academic center to validate the principles of optical imaging for frozen section consultation. In this case study, NIR imaging was able to rapidly locate two pathologic lesions, after initial grossing under white light located only one. Smart-Cut therefore has the potential to speed up the FS consultation process, and possibly even identify lesions that would previously have been found only on permanent section.

The Smart-Cut system has several limitations. Smart-Cut for FS consultation secondary to intraoperative molecular imaging is limited by the efficacy of the targeting agent. The high PPV of Smart-Cut is due to dye localization to the tissue of interest. For example, molecular imaging with TumorGlow^®^ is highly effective for visualizing pulmonary metastases of sarcoma or colorectal carcinoma, but relatively ineffective for visualization of metastatic melanoma [9, 13, 16]. Consequently, if imaging exclusively with TumorGlow^®^, Smart-Cut is hypothesized to be ineffective for metastatic melanoma in the lungs, and for any lesion not receptive to the contrast agent. As such, Smart-Cut cannot currently be relied upon for an adequate negative predictive value. However, this shortcoming can be addressed by optimizing contrast agents used for each patient, based on clinical judgement, pre-operative imaging or tissue biopsy, and/or radiographic presentation to assure dye uptake in the lesion. Additionally, the use of multiple contrast agents in a “dye cocktail” would improve sensitivity of Smart-Cut. By using dyes of differing emission wavelengths, Smart-Cut can leverage the interchangeability of its SPIIF imaging platform to maintain receptor-specificity of each individual dye while still appreciating the improved sensitivity of a dye cocktail. It should be noted that our Smart-Cut prototype is currently used for research purposes only.
